# Health System–Level Barriers to Living Donor Kidney Transplantation: Protocol for a Comparative Case Study Analysis

**DOI:** 10.2196/44172

**Published:** 2023-03-07

**Authors:** Anna Horton, Katya Loban, Peter Nugus, Marie-Chantal Fortin, Lakshman Gunaratnam, Greg Knoll, Istvan Mucsi, Prosanto Chaudhury, David Landsberg, Michel Paquet, Marcelo Cantarovich, Shaifali Sandal

**Affiliations:** 1 Research Institute of the McGill University Health Centre Montreal, QC Canada; 2 Department of Family Medicine and the Institute of Health Sciences Education McGill University Montreal, QC Canada; 3 Centre de recherche du Centre hospitalier de l’Université de Montréal Montreal, QC Canada; 4 Division of Nephrology Department of Medicine Centre hospitalier de l’Université de Montréal Montreal, QC Canada; 5 Matthew Mailing Centre for Translational Transplant Studies Lawson Health Research Institute London Health Sciences Centre London, ON Canada; 6 Division of Nephrology, Department of Medicine Schulich School of Medicine and Dentistry Western University London, ON Canada; 7 Division of Nephrology Department of Medicine University of Ottawa Ottawa, ON Canada; 8 Clinical Epidemiology Program Ottawa Hospital Research Institute Ottawa, ON Canada; 9 Ajmera Transplant Center and Division of Nephrology University Health Network Toronto, ON Canada; 10 Division of Nephrology University of Toronto Toronto, ON Canada; 11 Department of Surgery McGill University Health Centre Montreal, QC Canada; 12 Department of Medicine University of British Columbia Vancouver, BC Canada; 13 Division of Experimental Medicine Department of Medicine McGill University Health Centre Montreal, QC Canada

**Keywords:** transplantation, living donor kidney transplantation, health systems, barriers, resource based theory, complex adaptive systems

## Abstract

**Background:**

Living donor kidney transplantation (LDKT) is the best treatment option for patients with kidney failure and offers significant medical and economic advantages for both patients and health systems. Despite this, rates of LDKT in Canada have stagnated and vary significantly across Canadian provinces, the reasons for which are not well understood. Our prior work has suggested that system-level factors may be contributing to these differences. Identifying these factors can help inform system-level interventions to increase LDKT.

**Objective:**

Our objective is to generate a systemic interpretation of LDKT delivery across provincial health systems with variable performance. We aim to identify the attributes and processes that facilitate the delivery of LDKT to patients, and those that create barriers and compare these across systems with variable performance. These objectives are contextualized within our broader goal of increasing rates of LDKT in Canada, particularly in lower-performing provinces.

**Methods:**

This research takes the form of a qualitative comparative case study analysis of 3 provincial health systems in Canada that have high, moderate, and low rates of LDKT performance (the percentage of LDKT to all kidney transplantations performed). Our approach is underpinned by an understanding of health systems as complex adaptive systems that are multilevel and interconnected, and involve nonlinear interactions between people and organizations, operating within a loosely bounded network. Data collection will comprise semistructured interviews, document reviews, and focus groups. Individual case studies will be conducted and analyzed using inductive thematic analysis. Following this, our comparative analysis will operationalize resource-based theory to compare case study data and generate explanations for our research question.

**Results:**

This project was funded from 2020 to 2023. Individual case studies were carried out between November 2020 and August 2022. The comparative case analysis will begin in December 2022 and is expected to conclude in April 2023. Submission of the publication is projected for June 2023.

**Conclusions:**

By investigating health systems as complex adaptive systems and making comparisons across provinces, this study will identify how health systems can improve the delivery of LDKT to patients with kidney failure. Our resource-based theory framework will provide a granular analysis of the attributes and processes that facilitate or create barriers to LDKT delivery across multiple organizations and levels of practice. Our findings will have practice and policy implications and help inform transferrable competencies and system-level interventions conducive to increasing LDKT.

**International Registered Report Identifier (IRRID):**

DERR1-10.2196/44172

## Introduction

End-stage renal disease represents a major public health burden. Patients needing dialysis have extremely poor survival rates when compared with the general population [1,2]. This is because these patients have a higher cardiovascular disease burden, greater susceptibility to infections, and demonstrate a decreased response to vaccination [3-6]. The intrusiveness of dialysis can significantly impede normal facets of life, such as work, vitality, and freedom to travel [7].

Kidney transplantation, in particular living donor kidney transplant (LDKT), is widely regarded as the best therapeutic option for patients with kidney failure. When compared with patients undergoing dialysis, those who have undergone kidney transplantation experience a 64%-75% lower risk of death by the first year following transplantation [8-13]. LDKT is a surgery that involves a healthy individual donating one kidney to a patient (recipient) with kidney failure. Given that there is a finite pool of deceased donors in any given year, and that the demand for organs far exceeds its supply [11,14], pursuing LDKT can narrow this gap and provide early access to a transplant. There are also many medical benefits to LDKT. The median survival of a kidney transplant from a living donor is longer than that from a deceased donor [8-11]. Those with LDKT also experience lower rates of acute rejection, spend less time on dialysis, and have an improved quality of life [10,15-21]. Thus, there is considerable interest in increasing the rate of LDKT [15].

Despite its significant benefits, LDKT rates in Canada have stagnated over the past decade and continue to average around 12-14 living donors per 1 million population. This is despite national efforts to increase LDKT, such as the paired kidney exchange program [1,10,22,23]. There are also significant interprovincial variations across provinces. For example, in Quebec, Ontario, and British Columbia, Canada’s most populous provinces, <15%, 30%-40%, and 50%-60% of kidney transplants performed annually are from living donors, respectively [11]. Similar trends are noted when comparing the living donor rates per million population across these provinces [1,10,23,24]. The reason for this significant disparity in a country with universal health care is not known.

Currently, the impetus of finding living donors is largely placed on the patient, and much of the present work to increase LDKT focuses on patients and addressing these microlevel barriers [25]. We conducted a qualitative study, exploring the perspectives of health professionals on the provision of LDKT [26,27] and identified poor communication between treating teams, absence of consistent guidelines, and lack of resources as barriers. Notably, some of these barriers were more prominent in provinces of Canada that have lower rates of LDKT. This work alluded to the existence of systemic attributes that impede the effective delivery of LDKT, thus driving the need to understand the factors driving these differences to inform system-level interventions to increase LDKT.

As such, the objective of the study described in this protocol is to generate a systemic interpretation of LDKT by identifying the attributes and processes that facilitate the delivery of LDKT in a provincial health system and those that create barriers. We also aim to identify the differences between these attributes and processes by comparing higher- and lower-performing systems. These objectives are contextualized within our broader goal of increasing rates of LDKT in Canada, particularly in lower-performing provinces. Our primary research question is the following: what are the attributes and processes of provincial health systems that account for variability in LDKT rates?

## Methods

### Research Approach

**Figure 1:**
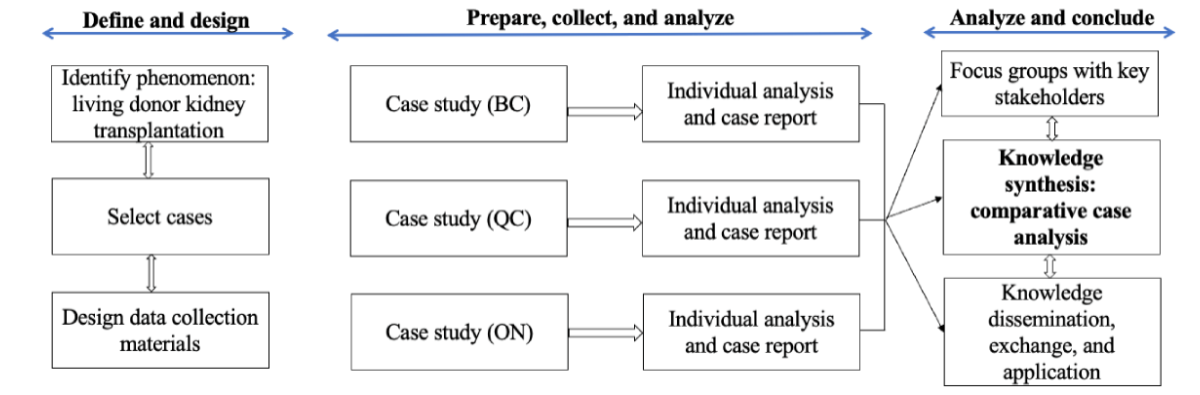
Comparative case study analysis design (adapted from Yin’s [28,29] methods of case study research). BC: British Columbia; ON: Ontario; QC: Quebec.

This study takes the form of a comparative case study analysis as described by Yin [28,29] and illustrated in Figure 1. Case study research is an in-depth, noninterventional examination of a single case over time to investigate a contemporary phenomenon in its natural context [29,30]. Case studies have been identified as the preferred methodology for examining high-performing health systems [31-39]. As quantitative data concerning rates of LDKT across provinces have been well-documented elsewhere [40-4], our study follows a qualitative design to investigate *how* and *why* these differences in performance exist. Qualitative methods have established relevance to answering these questions in health research [43]. Understanding the system as an integrated whole lies in understanding the patterns and relationships between its levels and key players [44]. Our qualitative design will investigate these real-world behaviors and perspectives at multiorganizational levels, in order to understand full system function. Thus, our case study analysis will be explanatory because it will explore and connect how certain attributes and processes of a provincial health system are linked to the provision of LDKT [28]. It will be inductive—that is, allowing themes and explanations to be derived primarily from a close reading of the data—without trying to fit it into a priori concepts.

Conceptually, our approach is underpinned by an understanding of health systems as complex adaptive systems (CASs). The concept of CAS stems from the complexity theory and takes a dynamic systems approach. A CAS is “an entity composed of many different parts that are interconnected in a way that gives the whole capabilities that the parts don’t have on their own” [45]. A provincial health system that delivers LDKT can be understood as a CAS, in that, it is a multilevel, interconnected system that involves nonlinear interactions between people and organizations, operating within a loosely bounded network. CAS approaches have been used increasingly as an analysis and research development tool in health care, with favorable results [44,46,47].

There also exists a tight fit between a CAS approach and case study methodology [44]. Researchers have identified that multiple methods that are often used in case study research lend themselves to understanding emergent elements and system dynamics [44]. Complexity theory also suggests that comparing the best to the worst in multiple case comparison can be a fruitful way of understanding the source of new structural arrangements and patterns of behavior [44,48]. Accordingly, our comparative method entails the comparison and synthesis of the similarities, differences, and patterns across multiple cases that share a common goal [39,49].

### Case Definition

**Figure 2:**
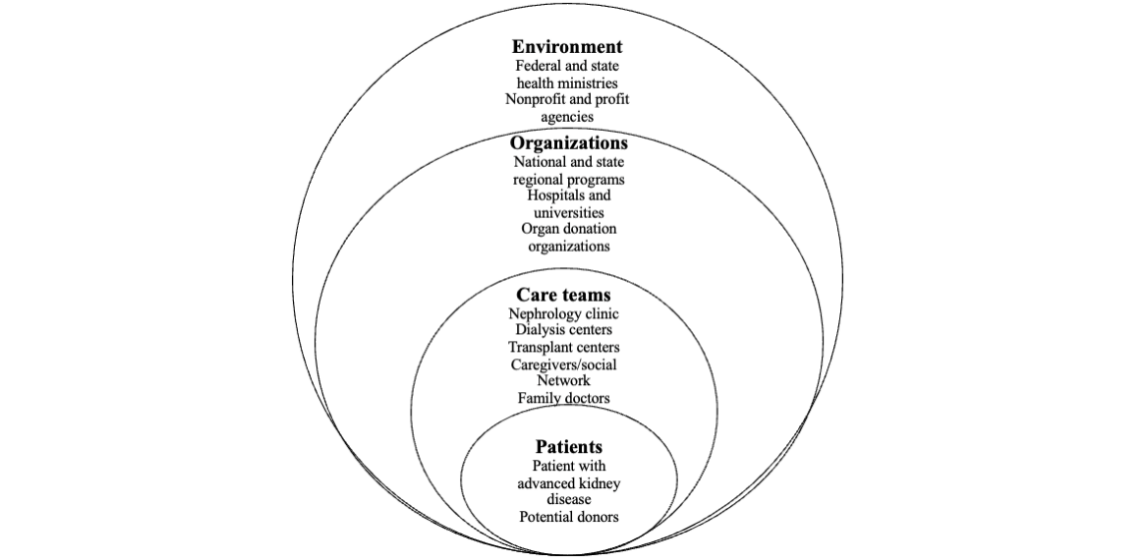
Envisioning the health system that delivers living donor kidney transplantation to patients as a complex adaptive system (adapted from the 4-level model proposed by the National Academy of Engineering [United States] and Institute of Medicine [United States] Committee on Engineering and the Health Care System).

In accordance with the CAS theory, we defined each provincial “case” as the health system involved in facilitating LDKT. Adapted from the 4-level model proposed by leading agencies [50] we mapped a whole-system model of LDKT, composed of macro-, meso-, and microlevels of practice that are interconnected, dynamic, and nested, with the patient at its core (Figure 2) [51]. These levels include organizations, service providers, recipients, and donors, representing the human and nonhuman agents that are implicated in the delivery of LDKT and thus form the elements for our analysis.

### Case Selection

We will conduct a comparative case study between British Columbia, Ontario, and Quebec, which represent respectively high, moderate, and low performance in LDKT as defined by the percentage of LDKTs to all transplantation performed annually [11]. These three provinces represent 75% of Canada’s population, and over 70% of the patients with end-stage renal disease reside in these provinces. Thus, performance in these provinces significantly influences the country’s overall transplant results. Additionally, since these provinces have low, moderate, and high rates of LDKT, we have an ideal case mix to facilitate interprovincial learning, as well as for theoretical replication, reliability, and external validity [28].

### Design

Our study follows sequential stages of data collection and analysis (Figure 1). We have conducted independent case studies of the health systems in British Columbia, Ontario, and Quebec using the data collection methods discussed below. Following data collection and independent analysis in all 3 provinces, we have recently conducted focus groups with stakeholders from across Canada, asking their opinions and experiences of the themes derived from the preliminary analysis of these cases. We will now conduct a comparative case study of British Columbia, Ontario, and Quebec. We will use the focus group data to develop and refine the themes from our comparative case study to form a final analysis.

### Participants

Participants from different levels of the health system, as shown in Figure 2, were recruited for interviews. As LDKT is organized largely by each province, representatives from federal bodies were invited to participate in focus groups, where we discussed and refined the national relevance of our findings. Table 1 shows a breakdown of participants invited for an interview. The composition of interviewees seen in Table 1 comprises an approach to “studying through,” tracing relations among actors, institutions, and discourses across spaces through interview data [52,53]. In accordance with our CAS approach, our participants represent macro-, meso-, and microlevels of practice. As the organizations implicated in LDKT vary among British Columbia, Ontario, and Quebec—for example, Quebec does not have a provincial renal program—the list of participants invited was adjusted accordingly.

**Table 1:** Types of participants and the numbers that were targeted in each category in each province.

Participant category	Number for each province
Ministry of Health representative	1-2
Organ Donation Organization representatives	2-3
Renal program representatives	2-3
Health care professionals at transplant centers	8-10
Health care professionals at nephrology clinics or dialysis centers	8-12
Living donor kidney transplant recipients	2-4
Living donors	2-4

### Recruitment

To recruit participants, purposive criterion sampling was used to invite key leadership at organ donation organizations, provincial renal programs, and transplant centers. Participants were considered to have key leadership roles if they held decision-making authority with interorganizational impact. Thereafter, snowball sampling was used to recruit providers from kidney care clinics and dialysis centers [52,55]. Data gathering continued until data saturation was reached; that is, when new interviews and document reviews did not provide additional information [56].

### Data Collection Methods

#### Semistructured Interviews

Semistructured interviews were conducted to understand the dynamic organization, governance, and care entailed in LDKT delivery and the interdependencies between the elements of each provincial health system. We also sought to understand what aspects of the system variously promoted or hindered patient access to LDKT. Interview guides for professional participants addressed their involvement in facilitating LDKT for patients, their interactions with other professionals in this process, their attitude toward LDKT, and which phenomena helped and which ones posed challenges in their work. Interview guides for donors and recipients of LDKT focused on their experiences of LDKT, their perception of care, and what helped and hindered their care path. Distinct interview guides with open-ended questions were developed for each category of participant with the combined expertise of our research team and preliminary document review (see sample guide in Multimedia Appendix 1). We followed an iterative approach whereby issues or ideas identified by participants were discussed with subsequent participants to enable further definition and refinement of themes [57]. Interviews were conducted remotely in English or French by our bilingual research coordinator (AH). All interviews were digitally recorded and transcribed. Participants were compensated with a CAD $50 (US $37.57) gift card following their participation in the interview.

#### Document Review

Document review served as complementary data collection to inform our understanding of programs, policies, and resources concerning LDKT in each province and as means of triangulation with interview data [58]. Documents for review were identified in consultation with our collaborators in each province, during interviews, and using web searches of governmental, organ donation organizations, renal programs, and hospital platforms. Documents were included if they were a policy, guideline, resource, program outline, presentation, announcement, or report pertaining to LDKT. Searches were conducted in both English and French.

#### Focus Groups

Following data collection in all 3 provinces and the initial coding of individual case studies, we conducted 4 focus groups remotely with the purpose of gleaning opinions about our preliminary themes from key stakeholders (Table 2). Focus groups comprise a small group of people brought together to discuss a particular issue, under the direction of a facilitator [59]. They are widely used in health research and are recognized to produce considerable information in a short space of time [59]. We recruited previous interview participants for focus groups, as well as patients, patient partners, and professionals from other provinces of Canada. In these focus groups, we presented themes from our preliminary analysis of the 3 case studies to participants and asked them about their opinions and experiences. Focus group guides were developed from our initial data analysis and reviewed by the research team. Each focus group lasted approximately 60 minutes, comprised 5-13 participants, and was conducted by the research team experienced in facilitating discussions in this setting (AH and KL). The focus groups were audio-recorded and transcribed. Participants were compensated with a CAD $50 (US $37.57) gift card following their participation in the focus group.

**Table 2:** Types of participants who were identified for participation in the focus group.

Focus group	Approximate number of participants	Participant types	Language of conduct
1	10-12	A health care professional working in transplantation or nephrology; or a representative from a provincial renal program, organ donation organization, or provincial health ministry, who has previously participated as an interviewee in this study	English
2	4-6	A health care professional working in transplantation or nephrology; or a representative from a provincial renal program, organ donation organization, or provincial health ministry, who works in a province outside of British Columbia, Ontario, and Quebec	English
3	4-6	An LDKT^a^ recipient or living donor who has experienced LDKT in the last 7 years and whose preferred language of conduct is English	English
4	4-6	An LDKT recipient or donor who has experienced LDKT in the last 7 years and whose preferred language of conduct is French	French

^a^LDKT: living donor kidney transplantation.

### Data Analysis

#### Individual Case Studies

Data from each case study were analyzed using inductive thematic analysis [43]. Thematic analysis involves identifying and analyzing patterns of meaning [60], and mapping regularities and variations across different accounts [59,61]. It is best suited to studying the processes and attributes of a system because it provides a “map” of the content and patterns across a data set [59], preserving our whole-system approach to understanding LDKT delivery [44]. Interview transcripts were analyzed independently by 2 research associates experienced in qualitative research (AH and KL). NVivo (version 12; QSR International) was used to support data management and analysis.

Transcribed interview data were read and highlighted line-by-line to openly derive preliminary codes that emerged iteratively from the data set. These codes were organized into categories and subcodes to form an initial coding scheme. Codes were then compared across the data set for regularities and divergences and modified accordingly. Through this process of inductive analysis, a coding scheme evolved, which retained strong links with the original data set [62]. The resultant codebooks for each individual case study have been retained as the basis for cross-comparative analysis. Coding, emerging themes, links between themes, and any disagreements between the 2 research associates were discussed at regular research team meetings. Our analytic procedure of documents entailed appraising and synthesizing the data contained in documents, followed by clustering the documents thematically [58]. The 2 research associates compared interview data to verify and corroborate findings from each case study. Analyses from each province are being published as individual case studies as the research progresses.

#### Comparative Case Analysis

Our comparative analysis will operationalize the resource-based theory (RBT) to compare case study data and generate explanations for our research question. The RBT is a strategic management theory that provides a framework for explaining and predicting the basis of an organization’s competitive performance and advantage [63]. RBT involves a broad classification of resources as tangible and intangible assets and aims to assess how resources create strategic advantage by examining how they are combined and managed [64,65]. It is a framework with established relevance in health systems and health management research to understand problems of high organizational complexity [66]. Thus, there is a high level of theoretical congruence between the complexity theory and the RBT, as the RBT engages with the social complexity of how resources—physical, human, and organizational—are combined [67]. The importance of network competence, dynamic capabilities, and strategic alliances between organizations is also well-recognized in this framework for achieving strategic advantage [67-69]. This is particularly pertinent for the study of the CASs that provide LDKT, where management is distributed between organizations and interorganizational relationships [69]. As such, the RBT provides relevant and useful concepts to understand the full-system function of LDKT, as well as identify the attributes and processes that characterize a high-performing system. Our analysis will follow the principles of RBT, with an emphasis on collaborative organizational relationships, in order to understand and compare the whole-system function of LDKT provision.

Following inductive coding and individual analysis of data collected from British Columbia, Ontario, and Quebec, we will use an RBT framework to analyze and compare our case study data and generate explanations for our research question. To do this, we will organize codebooks from each province into capabilities identified from the RBT literature, following questions stemming from these capabilities to guide our organization (Table 3). According to the RBT, a capability is what can be done as a result of resources working together [70]. We will therefore delineate and compare the attributes and processes from each province that determine their capabilities to deliver LDKT. The same 2 research associates who coded individual case studies will go through codebooks from each case study to extract information about the RBT capacities identified above and assign existing codes to the relevant RBT capability, to build themes. Through an iterative process, we will assign codes to emerging themes, create new themes where needed, and merge themes if they replicate each other until saturation is reached. We will then compare and contrast the themes in each RBT capability among provinces to identify “distinctive competencies” [71]; that is, attributes and processes that exist in certain provinces and not in others. We will compare our focus group data (analyzed using inductive thematic analysis, following the same process described above [43]) to themes from the comparative analysis, adding these data to existing themes where it is concurrent and creating new themes where it diverges. This process will deepen our understanding of the attributes and processes we have identified, and their relevance to other Canadian provinces. Following this approach, we will develop explanations for how resources in each province are deployed to achieve strategic advantage and to explain one province’s strategic advantage relative to others in LDKT performance. We will contextualize these capabilities in characteristics described by interviewees that operate externally from the provincial organization of LDKT, which influence delivery. We will situate our analysis in a discussion of these external dependencies.

**Table 3:** Resource-based theory capacities for comparative analysis.

Capacity	Guiding questions
Resources	What, where, and how are resources deployed in LDKT^a^ delivery?
Competition for resources	What competition exists for resources to facilitate LDKT?
Organizational capacity	What are the organizational capacities of the organizations involved in LDKT delivery?
Collaborative capacity	What collaborative capacities exist in and between organizations?
Value creation	What activities create value for LDKT?
Dynamic capabilities	What are the dynamic capabilities of organizations?

^a^LDKT: living donor kidney transplantation.

### Ethics Approval

Ethics approval for this study was obtained from the McGill University Health Centre Research Ethics Committee (MP-37-2021-7126/LDKT Case Study). This study is being conducted in accordance with the Tri-Council Policy Statement: Ethical Conduct for Research Involving Humans (2014), and the Declaration of Istanbul.

## Results

This project was funded by a grant from a Gift of Life Institute, a Clinical Faculty Development Research Grant from the American Society of Transplantation from 2020 to 2021, and by a Health Research Grant from the Kidney Foundation of Canada. Individual case studies of British Columbia, Ontario, and Quebec were carried out between November 2020 and August 2022. The individual case study findings of LDKT delivery in British Columbia have been published [72,73]. Focus groups were carried out between June and November 2022. The comparative case analysis began in December 2022 and is expected to conclude in April 2023. Writing up of our findings is projected for May 2023, and manuscripts are expected to be submitted for publication by July 2023. A patient partner from Quebec was consulted regarding participant selection, study materials, and analysis and was involved in the publication of the case study [73].

## Discussion

### Expected Findings

This study aims to produce a system-level understanding of LDKT delivery in Canada’s 3 most populous provinces that have variable rates of LDKT, presenting a unique opportunity for comparative analysis. Informed by quantitative data [11], we are using qualitative methods to explain these differences. We want to identify the attributes and processes that facilitate or create barriers to LDKT using the RBT. Our preliminary findings suggest that barriers to effective LDKT delivery exist at different organizational levels of the health system and, critically, in the relationships between these organizations. LDKT delivery is aided by supportive governance organizations with a provincial overview, which increase the collaborative capacities of the system, boost organizational capacity, and generate value for LDKT across the different organizations involved [72,73].

To our knowledge, a comparative case analysis approach has not been used in the field of nephrology or kidney transplantation. Our approach has implications in these disciplines where there exists a poor understanding of system-level factors leading to inferior outcomes, inequities in access to therapy, and fractured transitions of care. This work also has global implications as LDKT is the main way to obtain a transplant in many countries that lack infrastructure for deceased donation. Based on our preliminary results and background work, we believe that to make significant improvements to LDKT delivery, interventions must target the dynamic relationships between different elements of a system. Much of the current work has focused on microlevel interventions to improve LDKT delivery [25,51], missing the important influence of meso- and macropractices and the dynamic interdependencies that exist between these levels of a health system [51]. Our findings will build on other studies that focus on implementing interventions to improve patient education and comfort on LDKT [16,74-78], and will help reenforce it by informing effective implementation strategies that encompass all levels of a health system.

### Limitations

The following limitations to our study may apply. First, our data collection largely pertains to 3 provinces of Canada and our findings may not be applicable to other regions and countries. Nonetheless, it should be noted that Quebec, Ontario, and British Columbia are Canada’s most populous provinces and represent 75% of the Canadian population, and our focus group data also go some way to establish the pertinence of our findings to other provinces. Our data also lay the foundations to extend our work across Canada and to other countries. Second, our research will not comprehensively explore the system-level factors leading to disparities in LDKT, such as gender and sex disparities and low rates of LDKT in Indigenous and other vulnerable populations. However, the data collected in this study will inform future systematic approaches needed to address this complex issue.

Another limitation may pertain to the challenge of delineating the “boundaries” of the health systems that form the basis for our cases. Identifying the unit of analysis for case study research has long been identified as a challenge [79], and in the complex field of health care, it may be difficult to differentiate between organizational and system boundaries and their environments [80]. We have delineated our cases by drawing on the extensive expertise of our team and collaborators, many of whom are practitioners in this field, as well as following a snowballing technique to iteratively identify the actors and organizations implicated in LDKT delivery. However, some perspectives may have been missed in this process, for example, family physicians who are primarily involved in living donor care for and may exercise influence over the LDKT process. To mitigate this challenge, we will situate our analysis in a detailed discussion of factors external to the boundaries of our case, which impact LDKT delivery. Where relevant, we will highlight areas for further research. Relatedly, there is some variation in our sample size and organizational representation between cases, given the heterogeneity in how care is structured in different provinces. This may have some impact on how we are able to compare across provinces. Our analysis will include an in-depth discussion of the structural differences in renal care among British Columbia, Ontario, and Quebec to situate our findings. Finally, though we believe our document review serves as useful complementary data collection and triangulation with interview data, we acknowledge that it may not be exhaustive.

### Conclusions

LDKT is the optimal treatment option for patients with kidney failure; yet, rates of LDKT have stagnated in Canada and vary significantly across provinces. There is a need to better understand how health systems deliver LDKT to patients. Following our prior work that has suggested system-level differences contributing to variability in LDKT performance, we will generate a systemic interpretation of LDKT delivery by identifying the attributes and processes that facilitate or create barriers to the delivery of LDKT. We will also identify the differences between these attributes and processes by comparing higher- and lower-performing provincial health systems. This qualitative comparative case study analysis is informed by CASs, and data analysis will be carried out in accordance with the RBT. Our findings will have practice and policy implications and help inform specific strategies, regulations, and infrastructure that are transferrable competencies and conducive to promoting the service delivery of LDKT. 

## Appendix

Multimedia Appendix 1 Peer-review report by La Fondation Canadienne du rein / Kidney Foundation - 2021 Kidney Health Scientific Committee (Montréal, Canada).

## Abbreviations

CAS: complex adaptive system
LDKT: living donor kidney transplantation
RBT: resource-based theory

## Data Availability

The data sets generated and analyzed during this study are not publicly available, as informed consent to share transcribed data for secondary use beyond this research was not obtained from the participants.
