# Supplementary material for: Health System–Level Barriers to Living Donor Kidney Transplantation: Protocol for a Comparative Case Study Analysis
Source: JMIR Res Protoc. 2023 Mar 7;12:e44172. doi: 10.2196/44172 (PMC10031444; doi:10.2196/44172)
Supplement: Multimedia Appendix 1 [file resprot_v12i1e44172_app1.pdf]

Applicant: Sandal, Shaifali
Title: Health System-Level Barriers to Living Donor Kidney Transplantation
Program: Kidney Health Research Grant
Institution: The Research Institute of the McGill University Health Centre
App #: 850317

View Review Information

Close Window Print

Committee: 2021 Kidney Health Scientific Committee (full proposal)

Scoring range for this committee: 5 Outstanding (Best) - 0.1 Poor (Worst)

Summary Statement

Overall Score: 4.106

Text Admin Summary:

Strengths:
-Application from a productive early career investigator who has identified an experienced collaborative team with a proven productivity in the research area.
-Previous criticisms have been addressed and the grant is improved.
-Proposed study is feasible in the hands of this team as the BC case study is already done, the study is supported by significant background work and addresses an important question relevant to the KFOC mandate.
-Good KT plan
Weaknesses:
-Only Minor issues were raised: Broad and non specific objectives identified although it is recognized that this is classic in qualitative research.
-Sex: not explicitly cited but will be incorporated in the data.

Reviewer Role: First Reviewer (528400)

Reviewer Summary: PI
Dr. Shaifali Sandal
Was appointed in January 2016, so not an early career investigator? Mitigating factor – had to become proficient in French before she could actually take up her clinical position. Her RI did allow her to start research activities on Jan 1, 2017.

Title
Health System-Level Barriers to Living Donor Kidney Transplantation
This is a re-application.
Career stage
***Assistant Professor, with 53 months on Faculty, therefore qualifies as an early career investigator (see above).
Prior grants held
PI: $50K from the American Society of Transplantation; $30K from the Canadian Donation and Transplant Research Program, $20K from McGill University Health Centre (MUHC)
CoPI/Co-app: 1 grant
Productivity
19 primary or senior author manuscripts; 1 book chapter; 13 first or senior author abstracts
Supervision
Primary supervisor for 6 post-graduate MD research projects
Background
Rates of living donor kidney transplantation vary across provinces in Canada. It is uncertain why this is the case. System-level differences in policy and practice in provinces may explain these disparate LDKT rates. A prior scoping review indicated that most of the literature on increasing LDKT consisted of individual-level interventions. A meta-analysis performed by the PI showed that most patient-level interventions were assessed by proxy outcomes and were of short duration. A qualitative study among health care practitioners in BC, ON and QC, identified 6 thematic barriers to LDKT but also brought to light the differences in health system organization WRT LDKT.
These provinces represent high, medium and low rates of LDKT – but the exact rate is only provided for BC (>= 20/million population) and a rationale for what constitutes high and low rates is not provided. A question would be the appropriateness of the denominator – looking at rates per 106 pop'n assumes that the proportion of potential recipients in the general population is the same in three Provinces. If Wouldn't a better denominator in the rate comparison be per patient-years of exposure to severe CKD (e.g. eGFR < 15 and or needing dialysis and with identified potential donors)?
Percentages of transplants that are from living donors are specified however, at 60% in BC, 30-40% in ON and < 15% in QC.
Hypotheses/Objectives
To determine how LDKT delivery is organized in three case provinces, what attributes of these systems facilitate or impede delivery of LDKT and what are the policies, regulations and infrastructure that underlie these systemic attributes.
Response to prior concerns
1. The original proposal focused only on system level factors and did not consider individual patient and provider factors that may be distributed differently in the studied provinces – the response indicates that all levels within health systems, including patient and provider, are explicitly considered in the proposed analysis
2. Describe how comparative case analysis been used in other disease states to assess changes in health systems – specific citation of the PIs own use of the method and references to other applications of the methods provided
3. There was discussion of gender categories but the gender groupings where not the same in each studied province – the PI points out that a comprehensive sex and gender-based analysis is not the primary focus of the proposed work and would necessitate a separate study
4. Lack of generalizability of the three selected Provinces to the rest of Canada – Provinces were selected according to LDKT rates to facilitate understanding of structural barriers and not necessarily to be representative
5. Not clear why two years of support would be required – addressed
6. Broad and non-specific objectives. Stated hypothesis is weak – the proposed research isn't meant to be hypothesis generating rather than hypothesis testing
7. Due to a large number of differences between Provinces, looking at a policy change in a single Province may be more productive – dynamic changes in policy will be considered
Methods
Three provinces with high (BC), moderate (ON) and low (QC) rates of LDKT have been identified. These three provinces constitute the majority of patients suffering from severe CKD in Canada.
Using web searches the investigators have mapped the operational framework for LDKT in each province. Data gathering

within each level of the LDKT delivery hierarchy (as shown in Fig2. Page 2 of the grant proposal document) will involve several elements:
Document review – using web searches and discussions with collaborators, the investigators have obtained critical documents relating to infrastructure, resources, programs, outreach, and personnel.
Interviews – with participants selected by purposive sampling (rather than random) which means that interviewees will be selected on the basis of their estimated ability to elucidate specific phenomena. Thereafter 'snowball sampling' will be used. The interviews will be digitally recorded and transcribed.
Field visits – direct observation of key meetings, educational sessions and other types of interactions.
Focus groups – with key national and provincial stakeholders (these will be conducted at the highest level of the hierarchy.
One issue is that the work appears to have already been performed in BC which, although bolstering confidence in the group being able to do the work, begs the question as to why the BC case study remains as the largest part of the project time line figure?
Analysis
Will follow an inductive framework. Data within each Province will be subject to thematic analysis using NVivo software to identify codes in the data and then cluster codes into themes.
Cross case analysis will be accomplished by comparing the content of themes across three Provinces.
Required expertise
Dr. Marie-Chantal Fortin, co-applicant - an expert in conducting qualitative analyses.
Dr. Peter Nugus, co-applicant - an expert in sociology, organizations, ethnography, interprofessional relations, identity and culture in complex organizations
Dr. Marcelo Cantarovich, co-applicant - Medical Director of Kidney Transplantation at the MUHC and a current president of The Transplantation Society and the former president of the Canadian Society of Transplantation
Dr. Istvan Musci, co-applicant – an expert on reducing barriers and disparities in access to LDKT
Dr. Greg Knoll, collaborator - expertise in quality metrics and measures in transplantation
Supporting publications = systematic review and meta-analysis of educational interventions to increase LDKT activity (published in Transplantation), book chapter in coordination of care in emergency departments, qualitative study of health professional-identified barriers to LDKT in BC, ON and QC (published in CJKHD), qualitative study of system-level barriers to LDKT in BC (submitted to CMAJ Open), survey of national transplant research program directors on patient engagement (published in Research Involvement and Engagement).
Infrastructure support
Research Institute of McGill University Health Centre
Budget
2 years
100K
79K for personnel costs and 21K for materials and supplies.
KT
End-of-grant workshops are planned
SGBA analysis
No at an explicit feature but sex/gender may fall out as a barrier/expediter at the patient level.
Ethics
Could not see REB approval nor the Informed Consent Form
Stakeholder priorities

Communication & connectivity - No
Health System approaches - Yes
Promoting Health & Quality of life - Yes
Keeping your Kidneys Healthy - No
Treatment of Kidney Disease - Yes

Reviewer Role: Second Reviewer (527956)

Reviewer Summary: Proposal is a resubmission to conduct a comparative analysis (qualitative) in 3 provinces (BC, Ontario, Quebec) to assess attributes/processes and barriers to LDKT. The 3 provinces have low, moderate and high rates of LDKT allowing for comparison.

Response to reviewers: excellent overall; added substantive changes to clarify the comparative case analysis methodology, previous uses and how the findings may be impactful.

Recent relevant research New investigator (initial 2016/1 with parental leave). PI: Hold small grants from AST, CDTRP; 22 publications since 2014

Statement of Objectives
Our specific research questions are the following:
- How is LDKT delivery organized in different provinces of Canada with variable performance?
- What current attributes and processes of a health system facilitate the implementation of LDKT for patients with kidney failure and what attributes and processes create barriers?
- What policies, regulations, and infrastructure are conducive to promoting the service delivery of LDKT to patients with kidney failure?

Our objective is to better understand the organization of LDKT delivery in different health systems with high, moderate, and low rates of LDKT, and synthesize this data to identify health system barriers and facilitators to LDKT. Our overarching goal is to increase LDKT in Canada.

Background Information and Literature Review
The rationale is well presented. The team has done extensive background work using multiple methods; they conducted a scoping/ sys rev of interventions; identified limitations of existing work; they conducted descriptive study of health providers as to what they perceive as barriers and identified interprovincial healthcare organization as a key to explaining the variability

Hypotheses and Research Questions
Our specific research questions are the following:
- How is LDKT delivery organized in different provinces of Canada with variable performance?
- What current attributes and processes of a health system facilitate the implementation of LDKT for patients with kidney failure and what attributes and processes create barriers?
- What policies, regulations, and infrastructure are conducive to promoting the service delivery of LDKT to patients with kidney failure?

Our objective is to better understand the organization of LDKT delivery in different health systems with high, moderate, and low rates of LDKT, and synthesize this data to identify health system barriers and facilitators to LDKT. Our overarching goal is to increase LDKT in Canada.

Design and Methodology
-hypothesis generating
-added more detail to how cases will be studied
-provided examples of previous use
-rationale for cases provided (provinces chosen)

-conducted background work mapping health systems in provinces of interest
- standardized document review data extraction forms may have been helpful
-plan to conduct document review, semi-structed interviews, field visits, focus groups then intra-and inter provincial analyses
- will follow standardized reporting
- initial work in BC started (intra-provincial analysis) with interesting findings; published in CMAJ OPEN

Ethical Issues
none

KNOWLEDGE TRANSLATION/IMPACT:
Innovative approach to a stagnant problem; addresses a priority area for the KF

BUDGET
Reasonable
